# The Function of MoGlk1 in Integration of Glucose and Ammonium Utilization in *Magnaporthe oryzae*

**DOI:** 10.1371/journal.pone.0022809

**Published:** 2011-07-27

**Authors:** Lisha Zhang, Ruili Lv, Xianying Dou, Zhongqiang Qi, Chenlei Hua, Haifeng Zhang, Zhengyi Wang, Xiaobo Zheng, Zhengguang Zhang

**Affiliations:** 1 Department of Plant Pathology, College of Plant Protection, Nanjing Agricultural University, and Key Laboratory of Integrated Management of Crop Diseases and Pests, Ministry of Education, Nanjing, China; 2 Biotechnology Institute, Zhejiang University, Hangzhou, China; University of Missouri-Kansas City, United States of America

## Abstract

Hexokinases are conserved proteins functioning in glucose sensing and signaling. The rice blast fungus *Magnaporthe oryzae* contains several hexokinases, including MoHxk1 (hexokinase) and MoGlk1 (glucokinase) encoded respectively by *MoHXK1* and *MoGLK1* genes. The heterologous expression of MoGlk1 and MoHxk1 in *Saccharomyces cerevisiae* confirmed their conserved functions. Disruption of *MoHXK1* resulted in growth reduction in medium containing fructose as the sole carbon source, whereas disruption of *MoGLK1* did not cause the similar defect. However, the Δ*Moglk1* mutant displayed decreased proton extrusion and a lower biomass in the presence of ammonium, suggesting a decline in the utilization of ammonium. Additionally, the *MoGLK1* allele lacking catalytic activity restored growth to the Δ*Moglk1* mutant. Moreover, the expression of *MoPMA1* encoding a plasma membrane H^+^-ATPase decreased in the Δ*Moglk1* mutant that can be suppressed by glucose and G-6-P. Thus, MoGlk1, but not MoHxk1, regulates ammonium utilization through a mechanism that is independent from its catalytic activity.

## Introduction

Glucose is a universal nutrient for most organisms and serves fundamental roles in energy supply, metabolism, development, cell proliferation, and even cell death. The ability to sense glucose is important for a broad range of organisms from bacteria and yeasts to plants and humans [1], [2], [3], [4], [5]. However, the mechanisms of glucose sensing and signaling to modulate the growth and development of organisms remain unclear.

In *Escherichia coli*, *Saccharomyces cerevisiae*, plants and mammals, hexokinases not only catalyze hexose phosphorylation as the first step of the glycolytic pathway, but also participate in glucose signaling [6], [7], [8], [9]. *S. cerevisiae* has three enzymes that catalyze hexose phosphorylation: the hexokinases (EC 2.7.1.1) Hxk1 and Hxk2, and glucokinase (EC 2.7.1.2) Glk1 [10]. Hxk2 plays a role in glucose-mediated catabolic repression [11]. Hxk2 was recently demonstrated to interact with the DNA binding transcription repressor Mig1 to generate a repressor complex that is translocalized into the nucleus. In the nucleus, the phosphorylation of serine 311 of Mig1 by Snf1 is essential for Mig1 protein nuclear export and derepression of gene expression in glucose-limited conditions. However, Hxk2 operates by interacting with both Mig1 and Snf1 protein kinase to inhibit Mig1 phosphorylation at serine 311 when glucose levels are high [12], [13].

The *Arabidopsis* genome contains two *HXK* and four HXK-like (HKL) genes [14], of which HXK1 is the glucose sensor that integrates nutrient and hormone signals to govern gene expression and plant growth in response to environmental cues [6], [15]. Moreover, genetic, biochemical, and molecular analyses have provided compelling evidence that *HXK1* forms the core of a glucose signaling complex with the vacuolar H^+^-ATPase B1 (VHA-B1) and the 19S regulatory particle of a proteasome subunit (RPT5B) in the nucleus to directly modulate specific target gene transcription that is independent of glucose metabolism [16].

Many functional hexose kinases have been identified in filamentous fungi. In *Aspergillus niger*, hexokinase and glucokinase contribute similarly to the rate of glucose phosphorylation *in vivo* [17], [18], whereas in *Neurospora crassa*, four different hexokinase isoenzymes have been described [19]. In addition, double mutants in both hexokinase and glucokinase genes of *Aspergillus nidulans* were unable to grow on either glucose or fructose. Hexokinase and glucokinase compensate each other for carbon catabolite repression by glucose in the single mutants, unlike *S. cerevisiae*, whose hexokinase and glucokinase play parallel roles in carbon catabolite repression by glucose [20], [21]. Nevertheless, it remains unclear how hexokinase and glucokinase participate in glucose repression. Recent studies have shown that the *Botrytis cinerea* hexokinase, but not glucokinase, is required for normal growth and sugar metabolism as well as pathogenicity in fruits, but neither hexokinase nor glucokinase is involved in carbon catabolite repression [22].

*Magnaporthe oryzae*, a filamentous ascomycete fungus, causes the most destructive disease of cultivated rice worldwide [23]. The appressorium, which generates turgor pressure, is necessary for effective infection by *M. oryzae* [24], [25], [26]. Many studies have focused on the regulation of appressorium formation and turgor pressure generation [23], [27], [28]. Tps1, which is involved in the establishment of plant disease, plays a pivotal role in the integration of glucose-6-phosphate (G-6-P) metabolism and nitrogen source use [29], [30]. Deletion of *tps1* in *M. oryzae* was defective in growth on glucose as the sole carbon source. The ability of *M. oryzae* Δ*tps1Δhxk1* mutants to grow in the presence of glucose was partially restored compared to Δ*tps1*. Furthermore, hexokinase activity and consequently the levels of G-6-P were reduced in Δ*hxk1* and Δ*tps1*Δ*hxk1* mutants [30]. Apart from these studies, however, surprisingly little is known about glucose sensing and signaling in *M. oryzae*.

Here, we report the identification of *M. oryzae MoGLK1* and *MoHXK1* genes by genetic complementation in a *S. cerevisiae* Δhxk1Δ*hxk2*Δglk1 triple mutant. Moreover, we demonstrate that MoGlk1, but not MoHxk1, plays a role in integration of ammonium and glucose utilization, but independent on glucokinase catalytic activity.

## Materials and Methods

### Fungal strains and growth conditions

*M. oryzae* wild -type strain Guy11 and mutant strains were cultured on CM medium [31] or MM medium [29] at 28°C. MM is 0.52‰ (w/v) KCl, 0.152‰ (w/v) MgSO_4_·7H_2_O, 0.152% (w/v) KH_2_PO_4_, 0.01‰ (w/v) thiamine and 0.1% (v/v) trace elements supplemented with 1% glucose or 1% fructose and 100 mM NaNO_3_ or 50 mM (NH_4_)_2_SO_4_ as different carbon or nitrogen sources, respectively.

### Cloning and sequencing MoGLK1 of and MoHXK1

The *M. oryzae MoGLK1* and *MoHXK1* open reading frames were amplified from cDNA, generated on RNA from mycelium of *M. oryzae* Guy11 with primers FL640(F)/FL641(R) and FL642(F)/FL1452(R) (Table S1), respectively. The amplified products were cloned into pMD19-T vector (TaKaRa, Dalian, China) and sequenced.

### Complementation of the Saccharomyces cerevisiae Δhxk1Δhxk2Δglk1 triple mutant

*MoGLK1* and *MoHXK1* were digested with *EcoR*I-*Pst*I and *EcoR*I-*Sal*I from pMD19 constructs, and subcloned into the pGBKT7 yeast expression vector digested with *EcoR*I-*Pst*I and *EcoR*I-*Sal*I, respectively. The vectors pGBKT7::*MoGLK1*, pGBKT7::*MoHXK1* and control vector pGBKT7 were transformed into the yeast Δ*hxk1*Δ*hxk2*Δ*glk1* triple mutant BY20022 (*MAT*a *his3*, *leu2*, *trp1*, *ura3*, *glk1*::*HIS3*, *hxk1*::*LEU2*, *hxk2*::*URA3*), kindly provided by Sakai Akira (Japan). Because the triple mutant strain does not have hexokinase activity, it can grow only on non-fermentable carbon source YPEG, containing 1% (w/v) yeast extract, 2% (w/v) peptone, 2% (w/v) ethanol, 2% (w/v) glycerol. For spot assays, the transformants were pregrown in SD+EG medium, which contains 0.17% (w/v) yeast nitrogen base (without amino acids), 5% (w/v) (NH_4_)_2_SO_4_, 2% (w/v) ethanol, 2% (w/v) glycerol, at 30°C for 48 h. After washing three times with ddH_2_O, aliquots (5 µl) of 10-fold serial dilutions were spotted on plates of SD medium, in the presence of 2% glucose (SD+Glc) or 2% fructose (SD+Fru) as the sole carbon source. Plates were incubated for 4 days at 30°C.

### Nucleic acid manipulation and Southern blotting

DNA and RNA extraction from *M. oryzae* were performed as described by Talbot *et al.* [31]. Gel electrophoresis, restriction enzyme digestions, ligations, DNA gel blot hybridization were performed using standard procedures [32]. Sequence alignments were performed using Clustal_W [33] and the calculated phylogenetic tree was viewed using Mega3.0Beta [34].

For Southern hybridization analysis to confirm *MoGLK1* and *MoHXK1* replacement, *Hind*III-digested genomic DNA from hygromycin-resistant Δ*Moglk1* and Δ*Mohxk1* transformants and wild type strain Guy11 was electrophoresed, blotted and hybridized with *MoGLK1* and *MoHXK1* probes, which were amplified from genomic DNA using the primers FL1359(F)/FL641(R) and FL1360(F)/FL1452(R) (Table S1), respectively. The probes were labeled with a Digoxigenin high-prime DNA labeling kit (Roche, Germany) and hybridization was carried out in accordance with the manufacturer's instructions. The blot was developed with the Digoxigenin detection starter kit 1 (Roche).

To detect *MoGLK1* and *MoHXK1* transcripts in the transformants, total RNA was isolated from mycelia cultured in CM liquid medium for 2 days. All RNA used for RT-PCR was treated with DNase I (TaKaRa, Dalian, China) prior to cDNA synthesis to exclude DNA contamination. First-strand cDNA was synthesized from the treated RNA using M-MLV Reverse Transcriptase (Invitrogen). Semiquantitative RT-PCR was performed on first-strand cDNAs. The primer pairs for transcript amplifications were as follows: *MoGLK1*, FL864(F)/FL641(R); *MoHXK1*, FL863(F)/FL1452(R); *ACTIN* (internal control), FL474(F)/FL475(R) (Table S1). To analyze the expression of the *MoPMA1* gene (MGG_04994), total RNA of wild-type strain Guy11 and Δ*Moglk1* mutant was isolated from mycelium cultured in 3-days liquid MM containing 1% glucose+100 mM nitrate, or 1% glucose+100 mM ammonium, 5% glucose+100 mM ammonium. RT-PCR was performed with primers FL1979(F)/FL1980(R) for *MoPMA1* and FL474(F)/FL475(R) for *ACTIN*. To determine the expression of *MoPMA1* in Δ*Moglk1* mutants transformed with wild-type *MoGLK1*, *MoGLK1^G69D^* and *MoGLK1^S156A^*, total RNA was isolated from those strains and the single strand cDNA was used for quantification. Real time PCR was performed with primers FL7272(F)/FL7273(R), and the transcript abundance relative to the constitutively expressed *ACTIN* gene was quantified. FL4362(F)/FL4363(R) was the primer pair of the *ACTIN* gene.

### Construction of gene deletion vector and fungal transformation

About 1-kb upstream and downstream flanking sequences of the *MoGLK1* gene were amplified by PCR, using genomic DNA as template, with primer pairs FL993(F)/FL1079(R) and FL1080(F)/FL998(R), respectively. The two PCR fragments were linked by overlap-PCR with primers FL993(F)/FL998(R), and the amplified products were cloned into pMD19-T simple vector (TaKaRa, Dalian, China) to form plasmid pMD::*MoGLK1*. A *Sma*I restriction site was incorporated into primers FL1079 and FL1080. The *HPH* gene expression cassette fragment was prepared by PCR with Primer STAR (TaKaRa, Dalian, China) from plasmid pCB1003 with primers FL344(F)/FL346(R), and then was inserted into the *Sma*I site of pMD::*MoGLK1* to generate the final construct pMD::*MoGLK1*::*HPH*. A 3.4 -kb fragment containing the deleted gene was amplified using plasmid pMD::*MoGLK1*::*HPH* as template with primers FL993(F)/FL998(R), purified by gel electrophoresis and used to transform protoplasts of *M. oryzae* strain Guy11. The construct pMD::*MoHXK1*::*HPH* was generated similarly with the primer pairs FL985(F)/FL1077(R) and FL1078(F)/FL990(R). Primer sequences are all shown in Table S1. Protoplast-mediated transformation of *M. oryzae* Guy11 was carried out following the method of Talbot *et al.* (1993).

### Targeted MoGLK1 and MoHXK1 gene replacement

To create *ΔMoglk1* and *ΔMohxk1* mutants, the 3.4-kb gene replacement fragment was introduced into protoplasts of Guy11, and hygromycin-resistant transformants were selected. Southern blots confirmed that *MoGLK1* and *MoHXK1* were successfully replaced in transformants G25 and G34 and H12 and H29 (Figure S1B), respectively. A single integration event had occurred in *ΔMoglk1* and *ΔMohxk1 mutant*s (not shown). RT-PCR was performed to further confirm that *MoGLK1* and *MoHXK1* gene replacement resulted in gene inactivation. The results showed the complete absence of *MoGLK1* or *MoHXK1* transcripts in the Δ*Moglk1* (G25 and G34) or Δ*Mohxk1* mutants (H12 and H29), respectively, as compared with constitutive *ACTIN* gene expression (Figure S1C).

### Complementation assays of catalytically inactive alleles of MoGLK1

For yeast complementation, site-directed mutagenesis was carried out by using plasmid pMD19::*MoGLK1* as template in the PCR reactions and with primers FL640(F)/FL2026(R), FL2027(F)/FL641(R) for the two fragments of mutant allele *MoGLK1^G69D^*, and FL640(F)/FL2028(R), FL2029(F)/FL641(R) for *MoGLK1^S156A^*, respectively. The two PCR fragments were linked by overlap-PCR with primers FL640(F)/FL641(R), the amplified products were digested with *EcoR*I-*Pst*I, and the resulting fragments were cloned into the *EcoR*I-*Pst*I sites of pGBKT7 to generate plasmids pGBKT7::*MoGLK1^G69D^* and pGBKT7::*MoGLK1^S156A^*, respectively. The mutants were confirmed by sequencing. Complementation assays of yeast Δ*hxk1*Δ*hxk2*Δ*glk1* triple mutant BY20022 was performed as described above.

For *M. oryzae* complementation assays, the promoter fragment of TrpC was cloned from pCB1003 plasmid by PCR using primers FL2500(F)/FL2501(R) and inserted into *Hind*III-*EcoR*I sites of pCB1532 [35] to produce vector pCB1532::PtrpC. The wild-type *MoGLK1* gene and the two catalytically inactive alleles *MoGLK1^G69D^* and *MoGLK1^S156A^* were amplified with primers FL640(F)/FL1000(R) by using pMD19::*MoGLK1*, pGBKT7-Mo*GLK1^G69D^* and pGBKT7-*MoGLK1^S156A^* as template, respectively. The products were digested with *EcoR*I-*Xba*I, and subcloned into the *EcoR*I-*Xba*I sites of pCB1532::PtrpC to generate the final vectors for *M. oryzae* transformation. Resistance to glufosinate ammonium (150 µg/mL) was used as a selectable marker during transformation, and transcripts of wild-type gene and catalytically inactive alleles were confirmed by semi-quantitative RT-PCR.

### Assays for vegetative growth and formation of conidia

Discs of mycelia, 5 mm in diameter, from 7-day-old CM plates were individually transferred to the center of 60 mm plates containing MM medium with 1% glucose or 1% fructose as carbon source, and with ammonium (1, 10, or 100 mM), nitrate (100 mM), or glutamate (100 mM) as nitrogen source. Photographs were taken after 3, 5, 7, and 9 days culturing at 28°C and the radial growth of vegetative mycelia was measured.

To induce production of conidia, mycelia were incubated on straw decoction (10%) and corn (4%) medium at 28°C in darkness for 7 days, followed by 3–4 days constant illumination [36], [37]. Conidia were harvested by washing with ddH_2_O, filtered through three-layer lens paper, concentrated by centrifugation (6,000 *g*) at 4°C for 10 min, and resuspended in 0.2 ml ddH_2_O. Conidia concentration was counted using a haemocytometer. Three plates were used for each strain and the experiments were repeated four times.

### Measurement of extracellular acidification and fungal biomass

Equal plugs of mycelia, from 7-day-old CM plates were transferred into liquid MM medium containing 100 mM ammonium and either 1% glucose or 1% fructose. The mycelia were cultured with shaking (125 rpm) at 25°C for 1, 2, 3, 5, 7 days. The initial pH of the medium was adjusted to a value of 4.5, 6.5, or 8.0. At each time point, mycelia were collected for biomass analysis and the pH of the remaining medium was measured. Fungal biomass was recorded by dry weight determination of three replicates per treatment in three independent experiments. For pH measurements, a model basic pH meter PB-10 (Sartorius, Germany) with a pH electrode was used.

### Qualification of ammonium

Equal plugs of mycelia, from 7-day-old CM plates were transferred into MM liquid medium with 0.1 mM, 1 mM and 100 mM ammonium respectively, and containing 1% glucose. The mycelia were cultured with shaking (125 rpm) at 25°C for 1, 2, 3, 5, 7 days as described above. At each time point, the medium was used to quantify the free ammonium. The ammonia assay procedure (Megazyme International Ireland Ltd., Ireland) and Spectrophotometer were used according to the manufacturer's instructions. The concentration of the remaining ammonium is calculated as follows, c = 0.07082×ammonium.

### Glucose-6-phosphate extraction and quantification

Glucose-6-phophate was extracted from the lyophilized mycelium according to Wilson *et al.* [30]. The quantification of glucose-6-phosphate was done by HPLC, using a Shodex KS-801 column (Tosho) and a refractive index detector. The running buffer was ddH_2_O. A column temperature of 50°C and a flow of 0.7 mL min^−1^ were routinely used. Glucose-6-phophate was purchased from Sigma.

### Quantitative RT-PCR

Total RNA was isolated from frozen fungal mycelia cultured in three different liquid media (CM, MM+1% glucose+100 mM (NH_4_)_2_SO_4_, and MM+1% glucose+100 mM NaNO_3_) with TRIzol Reagent (Invitrogen, USA) and the first- strand cDNA was synthesized with M-MLV Reverse Transcriptase (Invitrogen, USA) according to the manufacturer's instructions. Quantitative RT-PCR was run on the Applied Biosystems 7300 Real Time PCR System with SYBR *Premix Ex Taq*™ (Perfect Real Time, Takara, Japan). Normalization and comparison of mean Ct values were performed as described [38]. The experiment was conducted twice with three independent biological replicates.

### Measurements of glucokinase activity

Total cell protein was extracted from known amounts of dry mycelium using a modification of the method described by Cove [39]. Following grinding of mycelium in Eppendorf tubes using sterilized tooth picks, 1 ml of 0.1 M Na_2_HPO_4_, pH 7.5, was added, and the sample mixed briefly before centrifugation at 17,000 *g* for 5 min. Each sample was extracted in triplicate, and the supernatant stored at −80°C.

The glucokinase activity was measured at 22°C. All assay components were purchased from Sigma. Enzyme activities are expressed as the concentration of product formed in 1 min by total cell protein from 1 mg of mycelium. The glucokinase activity was determined by measuring the NADPH production at A_340_, at two time points. In these reactions, product formation is stoichiometric to changes in NADPH or NADP concentration. Protocols were obtained from www.sigma.com.

### Pathogenicity assay

Conidia used for pathogenicity assay were prepared from 14-day-old cultures and diluted to the concentration of 1×10^5^ spores ml^−1^ in sterile water supplemented with 0.2% (w/v) gelatin. Plant infection assays were performed on four-week old susceptible rice seedlings (*O. sativa*) CO-39 or seven-day old barley seedlings (Four arris) by spraying 4 ml of the conidial suspensions with a sprayer. Inoculated plants were placed in a moist chamber at 28°C for first 24 hrs in darkness, and then transferred back to another moist chamber with a photoperiod of 12 hrs under fluorescent lights [40], [41].

## Results

### Isolation and sequence analysis of MoGLK1 and MoHXK1

The *M. oryzae* genome contains a hexokinase gene (MGG_09289), *MoHXK1*, and a hypothetical glucokinase gene (MGG_03041), *MoGLK1* [42]. The glucokinase activity of MoHxk1 and its function in relation to Δ*tps1* was determined previously by Wilson *et al.* [28]. However, since full-length cDNA sequence of *MoHxk1* has not been described, we isolated full-length *MoGLK1* and *MoHXK1* cDNA from mycelia of *M. oryzae* wild-type strain Guy11 by RT-PCR. The cDNA fragments were cloned into plasmids and verified by sequencing to be identical to the sequences in the *M. oryzae* database (www.broad.mit.edu/annotation/genome/magnaporthe_grisea). The *MoHXK1* gene contains an ORF of 1434 bp and encodes a predicted protein of 52.5 kDa that shares 81% and 79% identity with *N. crassa* HXK and *B. cinerea* Hxk1, respectively (Figure S2). The *MoGLK1* gene contains an ORF of 1488 bp and encodes a predicted protein of 54.5 kDa that showed 60% identity to *B. cinerea* Glk1 (Figure S2). The phylogenetic tree of protein sequences of known hexose kinases revealed a clear cluster of fungal hexose kinases that is distinct from those of plants and animals (Figure S3). Among the fungal hexose kinases, MoGlk1 and MoHxk1 fall into the group of fungal hexokinases and glucokinases, respectively (Figure S3). Southern blot analysis of genomic DNA revealed that the two genes are both present in single copies in the *M. oryzae* genome (Figure S1B).

### Functional complementation in S. cerevisiae

To determine whether MoGlk1 has the hexose kinase activity, we tested the ability of *MoGLK1* to rescue the growth defect of the *S. cerevisiae* Δ*hxk1*Δ*hxk2*Δ*glk1* triple mutant BY20022 in the presence of hexose. We also included *MoHXK1* as control for this experiment. The coding regions of *MoGLK1* and *MoHXK1* were cloned into the yeast expression vector pGBKT7 and transformed into the yeast mutant BY20022. Because the yeast mutant has no hexokinase activity, it could only grow on non-fermentable carbon sources such as glycerol and ethanol. The functional expression of the *MoGLK1* and *MoHXK1* genes was tested by growing the yeast transformants on plates that contained glucose or fructose as the sole carbon source (Figure S4). Mutant yeast BY20022 cells transformed with either *MoGLK1* or *MoHXK1* cDNA could grow on medium containing glucose. However, only the transformant expressing *MoHXK1* was able to grow well on fructose, whereas *MoGLK1* did not support growth on fructose. The vector pGBKT7 alone was unable to restore growth of mutant BY20022 on either glucose or fructose medium. These data confirm that *MoGLK1* and *MoHXK1* encode a functional glucokinase and hexokinase, respectively.

### Phenotypic characterization of ΔMoglk1 and ΔMohxk1 mutants

To determine the functions of *MoGLK1*, deletion mutants were created by replacing the *MoGLK1* coding regions with the hygromycin phosphotransferase resistance gene (Figure S1A). To compare the functions of *MoGLK1* and *MoHXK1*, we also created the deletion mutants of *MoHXK1*. We investigated the role of *MoGLK1* and *MoHXK1* in the growth, development and virulence of *M. oryzae*. The Δ*Moglk1* and Δ*Mohxk1* mutants both showed normal mycelial growth on CM medium (not shown). However, sporulation of the Δ*Moglk1* mutant was dramatically reduced. The Δ*Moglk1* mutant produced only 9.67±2.31×10^3^ conidia (mean ± SE) per plate culture compared with 2.10±0.31×10^5^ for the Guy11 strain (*P*<0.01). Normal sporulation occurred in the *ΔMohxk1* mutant (not shown), which is in agreement with the result from Wilson *et al.* [30]. Conidial germination rates and appressorium formation were not affected in the Δ*Moglk1* and Δ*Mohxk1* mutants, and their ability to cause rice and barley blast disease was also not reduced (not shown).

Hexokinase is the first enzyme in the glycolytic pathway. To determine whether *MoGLK1* or *MoHXK1* are required for glycolytic regulation, the Δ*Moglk1* and Δ*Mohxk1* mutants were incubated on MM medium with nitrate as nitrogen source and with different carbon sources adjusted to pH 6.5 (Table 1). The Δ*Moglk1* mutants were indistinguishable from the wild-type Guy11 strain on all carbon sources tested. The Δ*Mohxk1* mutants grew well on most carbon sources, except on fructose. The diameter of the Δ*Mohxk1* mutants was ∼50% of that of Guy11 and Δ*Moglk1* mutants on fructose (Table 1). This is consistent with data from the triple mutant yeast complementation experiments, and shows that MoGlk1 provides the main source of glucokinase activity, while MoHxk1 provides glucokinase and fructokinase activities both in *S. cerevisiae* and in *M. oryzae*. However, Δ*Mohxk1* mutants still grew slowly on fructose (Table 1), probably because of the existence of other kinases that could also catalyze fructose.

**Table 1:** Growth of *ΔMoglk1* and *ΔMohxk1* mutants on different carbon sources.

MM +a	Guy11	Δ*Moglk1*	Δ*Mohxk1*
Glucose	2.43±0.06b	2.47±0.06	2.47±0.06
Fructose	2.37±0.12	2.47±0.12	1.21±0.05
Sucrose	2.43±0.06	2.53±0.15	2.47±0.15
Mannitol	2.60±0.10	2.67±0.12	2.53±0.06
Sorbitol	2.13±0.06	1.93±0.25	2.20±0.10
Sodium acetate	2.10±0.17	2.20±0.20	2.20±0.10

a MM, minimal medium containing 100 mM nitrate is described in materials and methods. The medium was supplemented (+) different carbon sources (1%) as shown. b Diameter of colonies (cm). All growth tests were carried out on agar-solidified medium and measured after 9 days incubation at 28°C.

### ΔMoglk1 mutants display decreased acidification in ammonium containing medium

**Figure 1:**
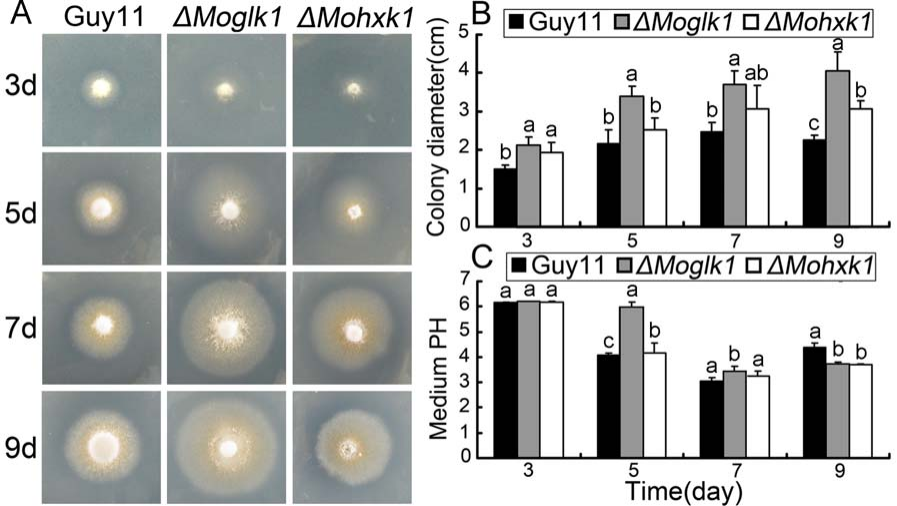
Ammonium utilization of Δ*Moglk1* and Δ*Mohxk1* mutants. Guy11, Δ*Moglk1* and Δ*Mohxk1 mutant*s were grown on MM containing 1% glucose and 100 mM ammonium. Photographs (A) were taken from one transformant of each mutant at indicated days after incubation at 30°C. Colony diameter (B) and medium pH (C) were measured at the same days. The experiment was performed with three strains for the Δ*Moglk1* and Δ*Mohxk1 mutant*s, respectively; and three independent replicates provided the same results. Different letter represent difference (P<0.05) among Guy11 and Δ*Moglk1*, Δ*Mohxk1*.

We observed the growth of *ΔMoglk1* and *ΔMohxk1* mutants on MM+glucose (GMM) or fructose (FMM) supplemented with 100 mM ammonium as the sole nitrogen source. The Δ*Moglk1* mutants grew equally well as Guy11 and *ΔMohxk1* on FMM with ammonium (not shown). On GMM with ammonium, however, Δ*Moglk1* mutants displayed larger colony after incubation for 9 days compared to Guy11 and *ΔMohxk1* mutants (Figure 1). Noticeably, Δ*Moglk1* mutants showed similar radial growth as Guy11 and Δ*Mohxk1* mutants for the first 5 days. However, the radial growth of Guy11 and Δ*Mohxk1* mutants on GMM with ammonium stopped after 5 days, whereas Δ*Moglk1* mutants were still able to grow for up to 9 days on this medium. Therefore, colonies of the Δ*Moglk1* mutants were eventually larger than those of Guy11 and the Δ*Mohxk1 mutant*s. To investigate the different growth ability of Guy11, *ΔMoglk1* and *ΔMohxk1* mutants on GMM with ammonium is not due to the high concentration of ammonium, we also tested their radial growth on GMM in different initial ammonium concentration (1 mM and 10 mM), of which Δ*Moglk1* mutants showed similar growth pattern compared to that with 100 mM ammonium, and larger colony after 9 days-incubation compared to Guy11 and Δ*Mohxk1* mutants (Figure S5).

**Figure 2:**
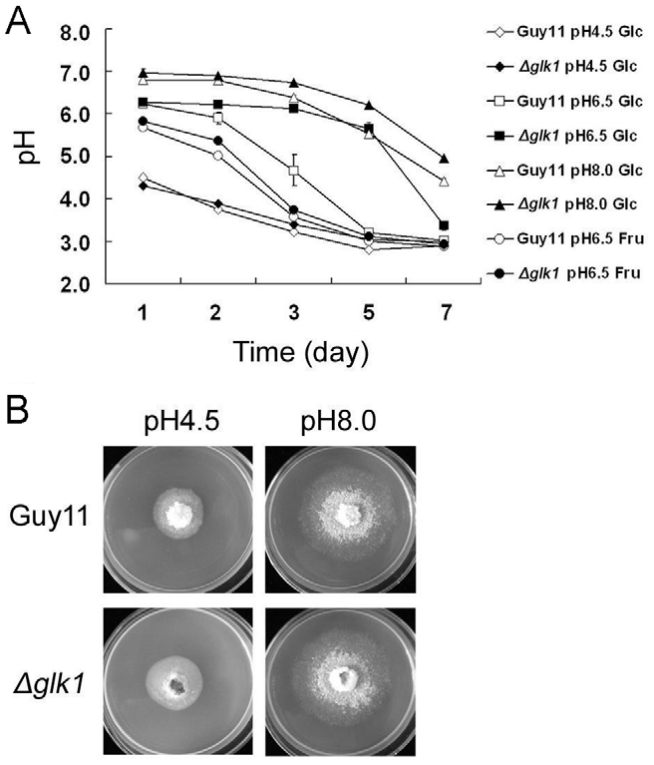
The Δ*Moglk1* mutants show reduced acidification on ammonium and glucose containing medium. (A) The effects of different sugars (1% glucose, or 1% fructose) and medium pH (4.5, 6.5, or 8.0) on extracellular acidification. The extracellular pH was measured in liquid cultures of wild type Guy11 and Δ*Moglk1* strains grown in the presence of 100 mM ammonium for various times, as indicated (days). The experiments were performed in six independent replicates. Error bars show the standard deviation of the mean for six replicates. (B) Guy11 and Δ*Moglk1* mutants were grown on MM containing 1% glucose and 100 mM ammonium agar plates with initial pH 4.5 or pH 8.0. Photographs were taken at 9 days after incubation at 30°C. The experiments were performed in triplicate.

Jernejc and Legiša [43] reported that the addition of ammonium to *Aspergillus niger* cultures results in rapid medium acidification. We thus considered the possibility that growth of *M. oryzae* on ammonium might lead to medium acidification, resulting in the inhibition of its own growth. To test this hypothesis, we monitored the extracellular pH in cultures of Guy11 and Δ*Moglk1* mutants grown in ammonium-containing GMM liquid medium. Initially, the medium was pH 6.5, but after incubation with Guy11 for up to 5 days, the extracellular pH dramatically decreased to 3.2. In contrast, when the Δ*Moglk1* mutants were grown in the same medium, the extracellular pH remained almost unchanged for 5 days, but on the 7th day, it also dropped to 3.3 (Figure 2A). Moreover, medium acidification was also observed in FMM, and Guy11 and Δ*Moglk1* mutants had roughly the same rate of acidification in the presence of ammonium, similar to the acidification by Guy11 in GMM (Figure 2A). The changes in the extracellular pH of *ΔMohxk1* mutants were similar to those of Guy11, regardless whether it was grown in GMM or FMM with ammonium (not shown). Furthermore, the drop in pH in cultures of Guy11 and Δ*Moglk1* mutants on agar plates was similar for media described above (Figure 1C). However, the growth of Δ*Moglk1* mutants on either GMM or FMM containing nitrate as nitrogen source didn't show any pH shift, which is the same as the growth of Guy11 and Δ*Moglk1* mutants (not shown). These results indicated that the growth of *M. oryzae* colonies was eventually inhibited because of the acidification of the medium. Δ*Moglk1* mutants showed a final larger redial growth in GMM with ammonium, resulting in a slower acidification than in Guy11 and Δ*Mohxk1* mutants.

To confirm that acidification is affected in Δ*Moglk1* mutants, we cultured Guy11 and Δ*Moglk1* mutants in GMM medium with ammonium adjusted to different initial pH. In medium with an initial pH of 4.5, the medium acidification (Figure 2A), the colony diameter was similar between Δ*Moglk1* mutants and Guy11 (Figure 2B), and the colony diameter of mutants trends to that of the Guy11 when grew in medium with an initial pH of 6.5. In medium with an initial pH of 8.0, the extracellular acidification in the culture of Guy11 was reduced (Figure 2A), and the radial growth of Guy11 increased relative to Δ*Moglk1* mutants (Figure 2B). Consistent with this, the diameter of colonies of Δ*Moglk1* mutants was similar to that of Guy11 when the medium was buffered to neutral pH (not shown). We conclude that the final larger redial growth of Δ*Moglk1* mutants on GMM with ammonium is due to a delay of extracellular acidification as compared to the wild-type strain.

### The ΔMoglk1 mutant displays decreased ammonium utilization

**Figure 3:**
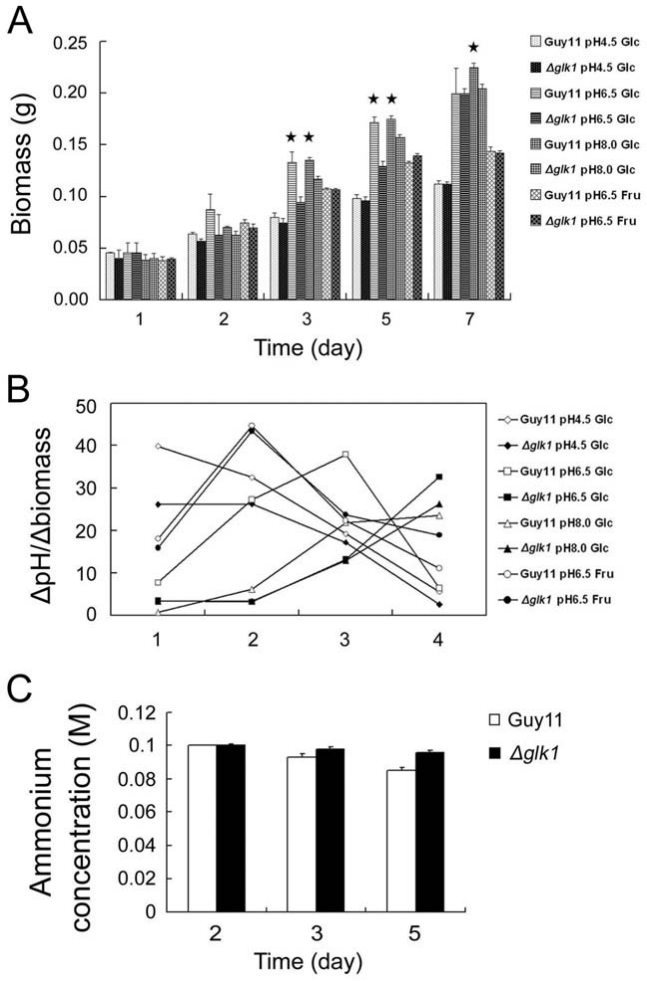
The Δ*Moglk1* mutants show decreased ammonium utilization in the presence of glucose as sole carbon source. (A) The dry weight of mycelia from Guy11 and Δ*Moglk1* strains grown in 100 mM ammonium-containing MM liquid culture with 1% glucose or 1% fructose as sole carbon source in indicated pH. Error bars indicate standard deviations from the mean. P values were calculated comparing Guy11 with Δ*Moglk1* mutant mycelia dry weight. * indicates P<0.05. (B) The relationship between the drop in extracellular pH and the increase of fungus biomass. X-axis represents the time interval. 1, between 2nd and 1st day; 2, between 3rd and 2nd day; 3, between 5th and 3rd day; 4, between 7th and 5th day. The Y-axis represents the ratio of pH and biomass. pH indicates the drop of pH between the interval days (data from fig. 2A); biomass indicates the increase of biomass between the interval day (data from fig. 3A). (C) The medium (MM+1% glucose) ammonium concentration after incubation of Guy11 and Δ*Moglk1* mutant for indicated days. The initial concentration of ammonium in the medium was 100 mM. Error bars indicate standard deviations from the mean. The experiment was repeated three times with similar results.

In *S. cerevisiae*, the pH of the culture can be used as a simple and effective control parameter for biomass-related products [44]. In some microorganisms, including yeast, a change in extracellular pH is correlated with ammonium assimilation [45]. Thus, we used MM liquid culture with ammonium to monitor the biomass of Guy11 and Δ*Moglk1* mutants over a period of 7 days. The Δ*Moglk1* mutants had significantly lower biomass than Guy11 after growth in neutral medium for 3 to 5 days and in alkaline medium for 3 to 7 days. In FMM medium or acidic GMM medium, the biomass was similar for Δ*Moglk1* mutants and Guy11 at all time points tested (Figure 3A). Furthermore, we monitored the relationship between the drop in extracellular pH and the increase of fungus biomass. The data shows that the slope of drop in pH/biomass of Δ*Moglk1* mutants slowed down especially in neutral GMM medium, and slowed down slightly in acidic and alkaline GMM medium compared to that of Guy11 (Figure 3B). These results indicate that ammonium utilization coincides with proton extrusion by *M. oryzae* and, secondly, that Δ*Moglk1* mutants might decrease ammonium utilization compared to Guy11 during growth on glucose-containing medium.

To confirm that ammonium utilization is reduced in Δ*Moglk1* mutants, the ammonium concentration was monitored in cultures of Guy11 and Δ*Moglk1* mutants incubated in GMM. The concentration of ammonium in the medium dropped more slowly in Δ*Moglk1* mutants than in Guy11 after five-days incubation (Figure 3C). This demonstrates that Δ*Moglk1* mutants decrease ammonium utilization in glucose-containing medium. Furthermore, to be sure that the defect of Δ*Moglk1* mutants is specific to the utilization of ammonium but not to the intermediates of ammonium catabolism, we investigated the growth ability of Δ*Moglk1* mutants on MM containing glutamate as the sole nitrogen source, which displayed similar radial growth of Δ*Moglk1* mutants and Guy11 (Figure S6).

### The regulation of ammonium utilization by MoGLK1 is independent of its catalytic activity

**Figure 4:**
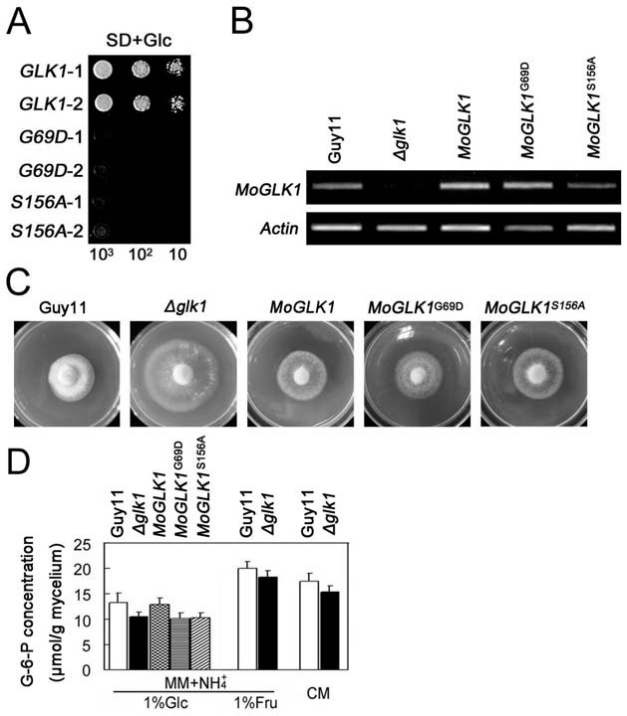
Catalytically inactive *Moglk1* alleles restore ammonium utilization in the *ΔMoglk1* mutant. (A) Complementation of yeast *Δhxk1*, *Δhxk2*, and *ΔMoglk1* triple mutant. Yeast cells transformed with *MoGLK1*, *MoGLK1^G69D^* and *MoGLK1^S156A^* were cultured in SD medium with glucose (Glc) as the sole carbon source. Photographs were taken after culturing at 30°C for 4 days. 1 and 2 represent two clones of each strain. (B) RT-PCR used to monitor the expression of *MoGLK1*, *MoGLK1^G69D^* and *MoGLK1^S156A^* in the *ΔMoglk1* mutant, using *ACTIN* as control. Total RNA was isolated from mycelium cultured in CM liquid medium for 2 days. The experiments were performed in triplicate. (C) The *ΔMoglk1* mutants transformed with wild-type *MoGLK1*, *MoGLK1^G69D^* and *MoGLK1^S156A^* were grown on MM agar plates containing 1% glucose and 100 mM ammonium. Photographs were taken at 9 days after incubation at 30°C. Three transformants for each strain were tested, and three experiments were performed and provided the same results. (D) G-6-P levels in mycelium of Guy11 and *ΔMoglk1* mutant and *ΔMoglk1* mutants transformed with wild-type *MoGLK1*, *MoGLK1^G69D^* and *MoGLK1^S156A^* in indicated medium conditions. Error bars indicate standard deviations from the mean. The experiments were performed in triplicate.

The glucose sensing activity of hexokinase in yeast requires catalytic activity [46], while in Arabidopsis it does not [6]. We set out to determine whether the catalytic activity of MoGlk1 is necessary to regulate ammonium utilization. Two conserved amino acid residues are essential for catalytic activity in yeast [46] and *Arabidopsis thaliana* hexokinases [6]. Targeted mutagenesis was performed to make Δ*Moglk1* mutant constructs that would encode enzymatically inactive proteins (Figure S1). In the *MoGLK1* ^G69D^ mutant, a glycine residue was replaced that eliminated the adenosine triphosphate binding function. In the *MoGLK1* ^S156A^ mutant, a serine residue was replaced that prevented phosphoryl transfer. However, both mutants retained the glucose-binding site [46]. Yeast functional complementation assays demonstrated that yeast BY20022 cells expressing either *MoGLK1* ^G69D^ or *MoGLK1* ^S156A^ did not grow on glucose as carbon source (Figure 4A). The wild-type *MoGLK1* and *MoGLK1* ^G69D^ and *MoGLK1* ^S156A^ alleles were then transformed into the Δ*Moglk1* mutant of *M. oryzae* to examine whether they could complement the mutation. The *Moglk1* transcript level in the transformants of the Δ*Moglk1* mutant complemented either with the wild type, the *MoGLK1* ^G69D^ or the *MoGLK1* ^S156A^ construct was similar, as detected by RT-PCR (Figure 4B). The constructs encoding catalytically inactive *MoGLK1* ^G69D^ and *MoGLK1* ^S156A^ alleles were similar to the wild-type *MoGLK1* construct in restoring the colony diameter of the Δ*Moglk1* mutant (Figure 4C), as well as in accelerating extracellular acidification of the *ΔMoglk1* mutant (not shown). At the same time, we tested the glucokinase activity and G-6-P leaves of Guy11, Δ*Moglk1* mutant and the Δ*Moglk1* mutants transformed with wild-type *MoGLK1*, *MoGLK1* ^G69D^ and *MoGLK1* ^S156A^. The glucokinase activities of the Δ*Moglk1* and the Δ*Moglk1* mutants transformed with *MoGLK1* ^G69D^ and *MoGLK1* ^S156A^ (1.8 nmol/min/mg mycelium) were lower than that of Guy11 and the Δ*Moglk1* mutants transformed with wild-type *MoGLK1* (6.5 nmol/min/mg mycelium). The levels of G-6-P were reduced in the Δ*Moglk1* mutant compared to the levels in Guy11 in all conditions (Figure 4D). Furthermore, the catalytically inactive *MoGLK1* ^G69D^ and *MoGLK1* ^S156A^ alleles, transformed into the *ΔMoglk1* mutant, could not restore the levels of G-6-P whereas the wild-type *MoGLK1* gene could restore the levels of G-6-P to those of the wild type Guy11 (Figure 4D). Considering that the glucokinase activities and G-6-P levels of the Δ*Moglk1* mutants and the Δ*Moglk1* mutants transformed with *MoGLK1* ^G69D^ and *MoGLK1* ^S156A^ might be produced by MoHxk1, these results are consistent with that MoGlk1 lacking the catalytic activity could still regulate ammonium utilization in glucose-containing medium.

### Genetic regulation of MoPMA1genes through MoGLK1

**Figure 5:**
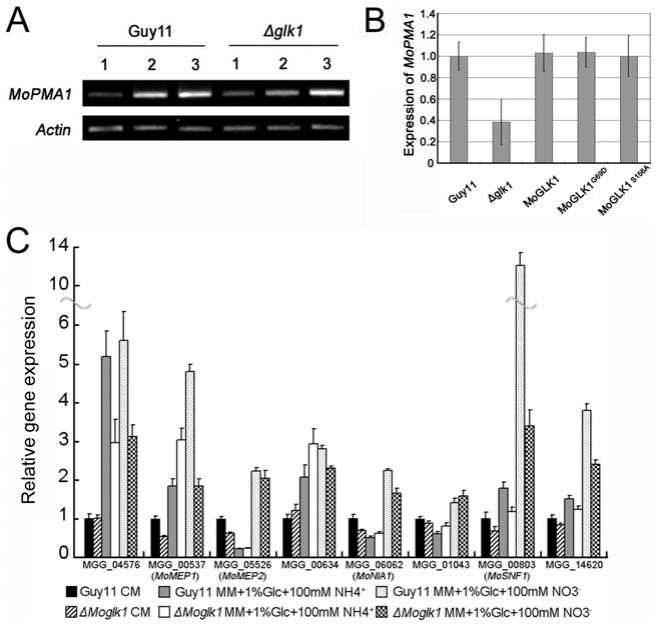
Gene expression analysis from Guy11 and Δ*Moglk1* mutants. (A) Expression levels of the *MoPMA1* gene in indicated conditions by semi-quantitative RT-PCR, using *ACTIN* as control. 1, MM+1% glucose+100 mM nitrate; 2, MM+1% glucose+100 mM ammonium; 3, MM+5% glucose+100 mM ammonium. (B) Relative transcript abundance of *MoPMA1* of Guy11 and Δ*Moglk1* mutant and Δ*Moglk1* mutants transformed with wild-type *MoGLK1*, *MoGLK1^G69D^* and *MoGLK1^S156A^* grown in MM with 1% glucose and 100 mM ammonium for 3 days. Each profile was normalized against *ACTIN* and compared relative to the expression of *MoPMA1* in Guy11. (C) qRT-PCR analysis of gene expression from Guy11 and Δ*Moglk1* mutant grown for 36 h in CM liquid medium and MM liquid medium containing either 100 mM ammonium or 100 mM nitrate as the sole nitrogen source. Gene transcript levels were normalized to the level of *ACTIN* and compared relative to the expression of each gene in Guy11 grown in CM. Error bars indicate standard deviations from the mean. Two independent biological repeats were performed and three technical replicates of each repeat were analyzed.

The plasma membrane H^+^-ATPase can transport protons out of the cytoplasm [47]. We therefore considered the possibility that a decrease in proton pumping in the Δ*Moglk1* mutant during growth on ammonium might result from repression of the *PMA* gene, which encodes plasma membrane H^+^-ATPase. To investigate this possibility, we identified a hypothetical gene of H^+^-ATPase, named *MoPMA1* (MGG_04994), present in a single copy in the genome sequence of *M. oryzae*. RT-PCR was performed to analyze the expression of *MoPMA1* in Guy11 and the *ΔMoglk1* mutant during growth on nitrate and ammonium (Figure 5). The transcript levels of *MoPMA1* were higher during growth on ammonium than on nitrate, both in Guy11 and the Δ*Moglk1* mutant (Figure 5A, compare lane 1 to lanes 2 and 3). However, the difference in transcript levels of *MoPMA1* between nitrate and ammonium was obviously lower in the Δ*Moglk1* mutant than in Guy11, during growth on ammonium with 1% glucose (Figure 5A, compare lanes 2, left and right panel, and B). Moreover, the catalytically inactive *MoGLK1* ^G69D^ and *MoGLK1* ^S156A^ alleles, as well as wild-type *MoGLK1* construct, transformed into the Δ*Moglk1* mutant, could restore the transcript level of *MoPMA1* to the level of Guy11 (Figure 5B). These results indicate that ammonium can induce high levels of *MoPMA1* transcript in *M. oryzae* and that MoGlk1 can modulate the expression of *MoPMA1* during the early stages of growth on ammonium, in a manner independent of its catalytic activity.

To investigate whether the reduced ammonium utilization in Δ*Moglk1* mutant is due to the decrease of transcript levels of ammonium transporter, we monitored the expression levels of *M. oryzae* ammonium transporter genes *MoMEP1* (MGG_00537), *MoMEP2* (MGG_05526) [48], and a predicted high affinity ammonium transporter gene MGG_04576 in CM liquid culture and MM liquid culture containing 1% glucose and 100 mM ammonium or 100 mM nitrate. The results showed that the transcript levels of *MoMEP1* and MGG_04576 were induced 3∼6 fold in MM containing either ammonium or nitrate compared to CM culture; and the transcript levels of *MoMEP2* was induced in MM containing nitrate (Figure 5C). However, the transcript levels of these putative ammonium transporter genes were constitutively similar between Guy11 and Δ*Moglk1* mutant in all the conditions tested (Figure 5C). This indicates that the deletion of *Moglk1* doesn't affect the expression levels of ammonium transporter genes, although the Δ*Moglk1* mutant showed reduced ammonium utilization.

Wilson and his colleagues found that MoTps1 integrates control of glucose-6-phosphate metabolism and nitrogen source utilization by sensing NADPH. *ΔMotps1* mutants do not express the nitrate reductase gene *MoNIA1* (MGG_06062) when exposed to nitrate, but have more G-6-P accumulation compared to wild-type strain [30], [48]. In Δ*Moglk1* mutant, G-6-P levels was decreased during growth in MM containing glucose compared with Guy11 (Figure 4C). Thus, we wonder whether the transcript levels of *MoNIA1* was increased in *ΔMoglk1* mutant compared with Guy11. To test this idea, we monitored the expression levels of *MoNIA1* and a predicted nitrite reductase gene MGG_00634, the results showed that both genes have similar expression levels between Guy11 and Δ*Moglk1* mutant in all the cultures tested (Figure 5C). Moreover, we monitored the expression levels of carbon catabolite derepression protein kinase genes *MoSNF1* (MGG_00803) [49] and MGG_14620, and a predicted catabolite repression gene MGG_01043, which showed similar expression pattern in Guy11 and Δ*Moglk1* mutant, respectively; excluding that *MoSNF1* was highly expressed in Guy11 compared to Δ*Moglk1* mutant in nitrate culture (Figure 5C).

## Discussion

Here, we report the characterization of *M. oryzae* MoGlk1 and MoHxk1, which are homologs to the hexose kinases from yeasts to mammals. The glucokinase activity of MoHxk1 and its function in relation to MoTps1 was determined previously by Wilson *et al.* [30]. However, we provide some more functional analysis of MoHxk1 in carbon and nitrogen sources utilization, which is distinguishable to MoGlk1. Complementation assays in *S. cerevisiae* indicated that MoGlk1 has a high affinity and a catalytic activity for glucose, whereas MoHxk1 can phosphorylate both glucose and fructose. These findings were confirmed in *M. oryzae*, because the Δ*Moglk1* mutant was able to grow on glucose and fructose in the presence of MoHxk1, whereas the Δ*Mohxk1* mutants grew very slowly on fructose. A similar phenotype has been described in *A. nidulans* [21]. In *B. cinerea*, the Δ*Moglk1* mutant was also found to grow normally, while the Δ*Mohxk1* mutant exhibited significant growth deficiency on several carbon sources, including fructose, sucrose, and sorbitol [22]. The *M. oryzae *Δ*Mohxk1* mutant does not display growth defects on other carbon sources apart from fructose, suggesting that sugar metabolic pathways in which hexokinases are involved apparently differ among fungi. It should be noted that the decline of G-6-P levels in the Δ*Moglk1* mutant was only 20% of that in Guy11, while in the Δ*Mohxk1 mutant* the decline in G-6-P was reported to be approximately fourfold, as compared to Guy11 [30]. This demonstrates that MoHXK1, but not MoGLK1, plays an important role in the generation of G-6-P in *M. oryzae*.

There were no obvious differences in the pathogenicity of Δ*Moglk1* and Δ*Mohxk1* mutants on barley and rice compared to Guy11 (not shown). The unaltered pathogenicity and nitrate utilization of the Δ*Mohxk1* mutant is same with that reported previously by Wilson *et al.* [30]. The functional analysis of *GLK1* and *HXK1* genes in *B. cinerea* showed a different result. Lesion formation and expansion of Δ*Bcglk1* and Δ*Bchxk1* mutants on tomato leaves were almost the same as the wild-type strain, whereas the Δ*Bchxk1* mutant (but not the Δ*Bcglk1* mutant) produced small lesions on tomato fruits and apples. The low virulence of the *B. cinerea* Δ*Bchxk1* mutant on fruit was proposed to be due to the high concentration of sugars in the fruits [22]. However, *M. oryzae* mainly infects leaves and stems of rice, in which concentrations of sugars are low. Thus, MoGlk1 and MoHxk1 appear not to be involved in pathogenesis in *M. oryzae*.

The growth of *M. oryzae ΔMoglk1* and Δ*Mohxk1* mutants on GMM with ammonium as the nitrogen source shows that Δ*Moglk1* mutants have lower ammonium utilization than do Guy11 and Δ*Mohxk1* mutants. However, the growth of *ΔMoglk1* and *ΔMohxk1* mutants on FMM with ammonium is similar to that of Guy11. These results suggest that MoGlk1 plays a role in the integration of ammonium and glucose utilization. In *S. cerevisiae*, Hxk2 is involved in glucose repression by sensing the glucose concentration [50], [51], [52] and deletion of the *HXK2* gene alleviated glucose repression, as evidenced by full respiratory growth at high glucose concentrations [53]. However, under nutrient starvation, the fermentative capacity of the Δ*hxk2* mutant is similar to that of the wild type [54]. Although there is no direct evidence that yeast *HXK2* regulates nitrogen source utilization, these studies suggest that *HXK2* may participate in the integration of carbon and nitrogen metabolism. A similar function has been reported in *Arabidopsis*, in which Hxk1 acts as a glucose sensor that integrates nutrient, light, and hormone signaling networks for the control of growth and development [6]. In *A. nidulans*, the Δ*hxk1*Δ*glk1* double mutant was strongly impaired in catabolite repression [21]. In *M. oryzae*, though the redial growth of the Δ*Mosnf1* mutant was reduced both on glucose containing medium and alternative-carbon-source media, it is likely that the growth defect in *M. oryzae* resulted from general vegetative growth defects via unknown mechanism by MoSnf1, directly or indirectly, and *MoSNF1* is dispensable for redial growth via carbon catabolite repression [49]. However, since reports of carbon catabolite repression in *M. oryzae* is rather limited, our results represent few examples where MoGlk1 might also be a glucose sensor that regulates ammonium utilization for vegetative growth.

The *Arabidopsis hxk1* mutant is insensitive to high concentration of glucose, and seedling development is not repressed by high concentrations of glucose [6]. However, we found that catalytically inactive alleles of both *MoGLK1* ^G69D^ and *MoGLK1* ^S156A^ could restore the ammonium utilization in the *ΔMoglk1* mutant but could not restore the levels of G-6-P and glucokinase activity. These data indicate that MoGLK1 can integrate the utilization of ammonium and glucose independent of catalytic activity and of the levels of G-6-P, which is consistent with the uncoupling of the sensing of glucose and glucose metabolism in *Arabidopsis* Hxk1 [6]. Therefore, we propose that MoGlk1 regulates ammonium utilization through sensing glucose that is independent on its catalytic activity. Moreover, neither *MoGLK1* ^G69D^ nor *MoGLK1* ^S156A^ could restore the sporulation as wild-type allele *MoGLK1* (not shown), further studies will need to elucidate the different mechanisms of MoGlk1 in regulation of ammonium utilization and sporulation.

Carbon and nitrogen sensing and signaling are important mechanisms that enable organisms to regulate metabolism and development. Organisms need to sense and integrate carbon and nitrogen metabolites to maintain their internal ratio of C to N in response to variation in nutrient status. *Arabidopsis* uses Hxk1 as a glucose sensor to interrelate nutrient, light, and hormone signaling networks to control growth and development in response to a changing environment [6]. In *S. cerevisiae*, TOR1 and SNF1, a cytoplasmic glucose sensor, both control the phosphorylation and cytoplasmic retention of GLN3, which is a GATA-type transcription factor of nitrogen catabolite-repressible genes. Therefore, glucose and nitrogen signaling pathways converge onto Gln3, which may be critical for both nutrient sensing and starvation responses [55]. We found that MoGlk1 could regulate ammonium utilization via the sensing of glucose. In *Arabidopsis*, sucrose can induce glutamine synthetase (GS), both at the level of mRNA accumulation and enzyme activity, to control ammonium assimilation [56]. In our study, we found that *MoPMA1* gene transcripts were lower in the Δ*Moglk1* mutant than in Guy11. It is indicated that MoGlk1 might regulate *MoPMA1*, transcripts to control ammonium utilization. Moreover, *M. oryzae* Tps1 can integrate G-6-P signals to regulate intracellular levels of NADPH and the expression of the nitrogen metabolite repressor gene *NMR1*, and thereby regulate the use of nitrate [30], [48]. In Δ*Motps1* mutant, G-6-P levels is accumulated and *MoNIA1* expression levels is abolished compared with Guy11. However, in our study, G-6-P levels is decreased and *MoNIA1* expression levels is similar compared with Guy11. This indicates that there are distinct regulation pathways of *M. oryzae* Tps1 in integrating G-6-P and nitrate utilization and MoGlk1 in integrating glucose and ammonium utilization. The molecular mechanisms linking MoGlk1, G-6-P, and MoPma1 still remain to be investigated, which should provides new insights in the integration of carbon and nitrogen sources in filamentous fungi.

## Supporting Information

Figure S1 **Targeted replacement of** ***MoGLK1*** **and** ***MoHXK1*** **.** (A) Organization of the *MoGLK1* and *MoHXK1* locus, before and after homologous recombination. The Orientation of *MoGLK1*, *MoHXK1* and *HPH* genes are indicated by black arrows. Upstream and downstream flanks of *MoGLK1* and *MoHXK1* are shown with grey boxes. The restriction sites indicated are for *Hind*III (H), *Pst*I (P), *Xho*I (X), and *EcoR*I (E). Scale bar = 1 kb. (B) Southern blots of DNA from wild-type strain Guy11 and selected transformants digested with *Hind*III and hybridized to a 989 bp fragment of *MoGLK1* and a 978 bp fragment of *MoHXK1*. Guy11 and putative transformants G2 and H9 contain the intact *MoHXK1* or *MoGLK1* genes. Transformants G25 and G34 or H12 and H29 do not hybridize to the native *MoGLK1* or *MoHXK1* genes, resulting from the gene replacement by the introduction of the hygromycin B -selectable marker. (C) Reverse transcript-polymerase chain reaction used to monitor the expression of *MoGLK1* in Guy11, G25 and G34 strains, and the expression of *MoHXK1* in Guy11, H12 and H29, using *ACTIN* as control. Gene replacement in G25 and G34 strains or H12 and H29 strains resulted in a complete loss of *MoGLK1* or *MoHXK1* transcripts.
(TIF)

Figure S2 **Alignment of** ***MoGLK1*** **,** ***MoHXK1*** **and hexokinases and glucokinases in other fungi.** The predicted amino acid sequences were aligned using Clustal W. The numbers indicated the amino acid residues. Gaps are indicated by dashes. Identical amino acids are highlighted on a black background, and similar amino acids on a light grey background. The hexokinase signature is marked by a single line. The black triangles indicate the key residues for catalytic activity.
(TIF)

Figure S3 **Phylogenetic analysis of** ***MoGLK1*** **and** ***MoHXK1*** **.** Dendrogram showing the relationship of mammalian, plant, insect and fungal hexose kinases based on amino acid sequences. Sequences were obtained from GenBank. Numbers after genes correspond to Genbank accession numbers. The phylogenetic tree was generated by the neighbor-joining (NJ) method using Mega3.0 Beta. Branch lengths are drawn to scale.
(TIF)

Figure S4 **Complementation of** ***S. cerevisiae*** **Δ** ***hxk1*** **Δ** ***hxk2*** **Δ** ***glk1*** **triple mutant.** Yeast cells transformed with plasmid pGBKT7 (control) containing *MoGLK1* or *MoHXK1* were cultured in YPEG medium to an OD600 of ∼1.0 at 30°C. Equal numbers of cells were spotted on YPEG or SD medium plates in the presence of glucose (Glc) or fructose (Fru) as the sole carbon source. Photographs were taken after culturing at 30°C for 4 days. 1 and 2 represent two independent yeast transformants containing *MoGLK1* or *MoHXK1* constructs.
(TIF)

Figure S5 **Radial growth of Δ** ***Moglk1*** **and Δ** ***Mohxk1*** **mutants on GMM with 1 mM (A, B, and C) and 10 mM (D, E, and F) ammonium.** Photographs (A and D) were taken from one transformant of each mutant at indicated days after incubation at 30°C. Colony diameter (B and E) and medium pH (C and F) were measured at the same days. The experiment was performed with three strains for the Δ*Moglk1* and Δ*Mohxk1 mutant*s, respectively; and three independent replicates provided the same results. Different letter represent difference among (P<0.05) among Guy11 and Δ*Moglk1*, Δ*Mohxk1*.
(TIF)

Figure S6 **Radial growth of Δ** ***Moglk1*** **and Δ** ***Mohxk1*** **mutants on GMM with 100 mM glutamate.** Photographs (A) were taken from one transformant of each mutant at indicated days after incubation at 30°C. Colony diameter (B) were measured at the same days. The experiment was performed with three strains for the Δ*Moglk1* and Δ*Mohxk1 mutant*s, respectively; and three independent replicates provided the same results. Different letter represent difference among (P<0.05) among Guy11 and Δ*Moglk1*, Δ*Mohxk1*.
(TIF)

Table S1 **Primer numbers and sequences used in this work.**
(DOC)
