# Supplementary figures and images for: The Function of MoGlk1 in Integration of Glucose and Ammonium Utilization in Magnaporthe oryzae
Source: PLoS One. 2011 Jul 27;6(7):e22809. doi: 10.1371/journal.pone.0022809 (PMC3144931; doi:10.1371/journal.pone.0022809)

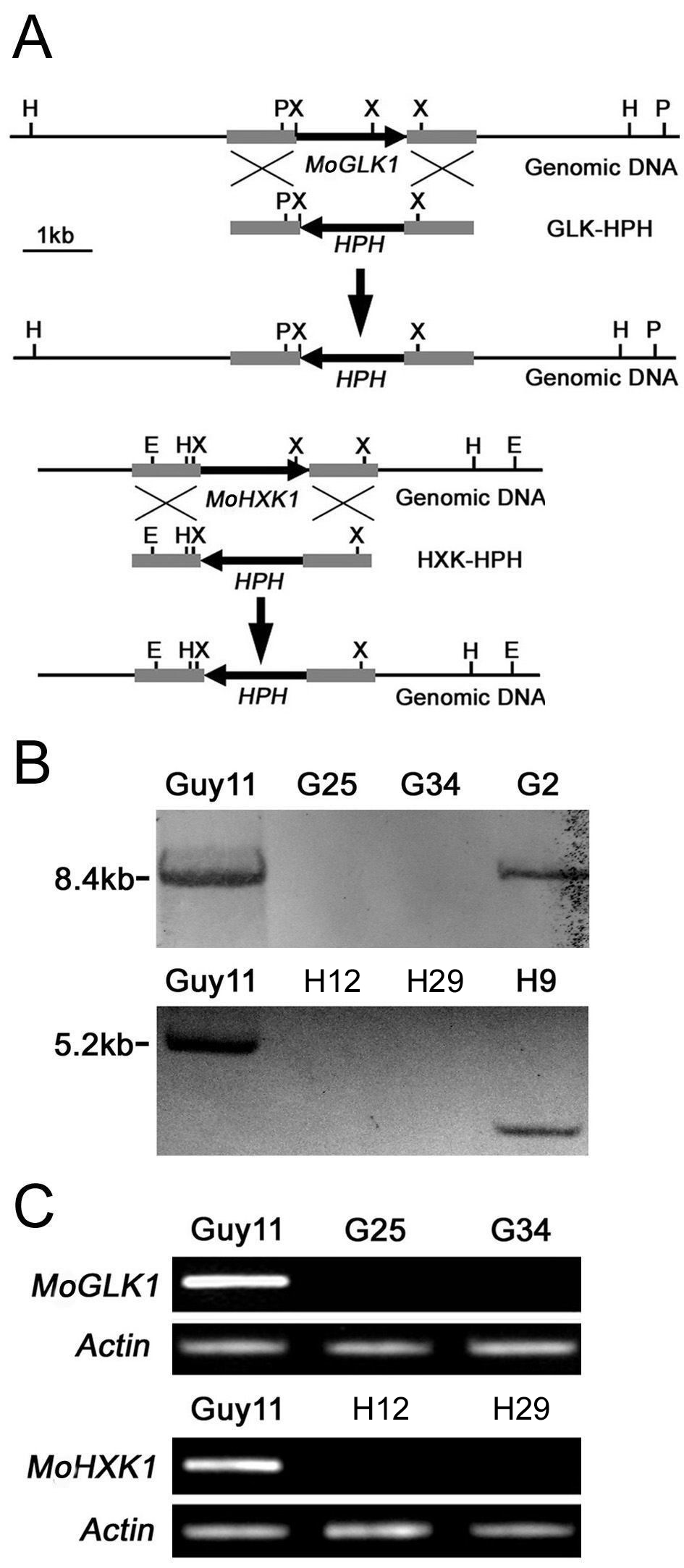

Supplement: Figure S1 — Targeted replacement of MoGLK1 and MoHXK1 . (A) Organization of the MoGLK1 and MoHXK1 locus, before and after homologous recombination. The Orientation of MoGLK1, MoHXK1 and HPH genes are indicated by black arrows. Upstream and downstream flanks of MoGLK1 and MoHXK1 are shown with grey boxes. The restriction sites indicated are for HindIII (H), PstI (P), XhoI (X), and EcoRI (E). Scale bar = 1 kb. (B) Southern blots of DNA from wild-type strain Guy11 and selected transformants digested with HindIII and hybridized to a 989 bp fragment of MoGLK1 and a 978 bp fragment of MoHXK1. Guy11 and putative transformants G2 and H9 contain the intact MoHXK1 or MoGLK1 genes. Transformants G25 and G34 or H12 and H29 do not hybridize to the native MoGLK1 or MoHXK1 genes, resulting from the gene replacement by the introduction of the hygromycin B -selectable marker. (C) Reverse transcript-polymerase chain reaction used to monitor the expression of MoGLK1 in Guy11, G25 and G34 strains, and the expression of MoHXK1 in Guy11, H12 and H29, using ACTIN as control. Gene replacement in G25 and G34 strains or H12 and H29 strains resulted in a complete loss of MoGLK1 or MoHXK1 transcripts. (TIF) [file pone.0022809.s001.tif]

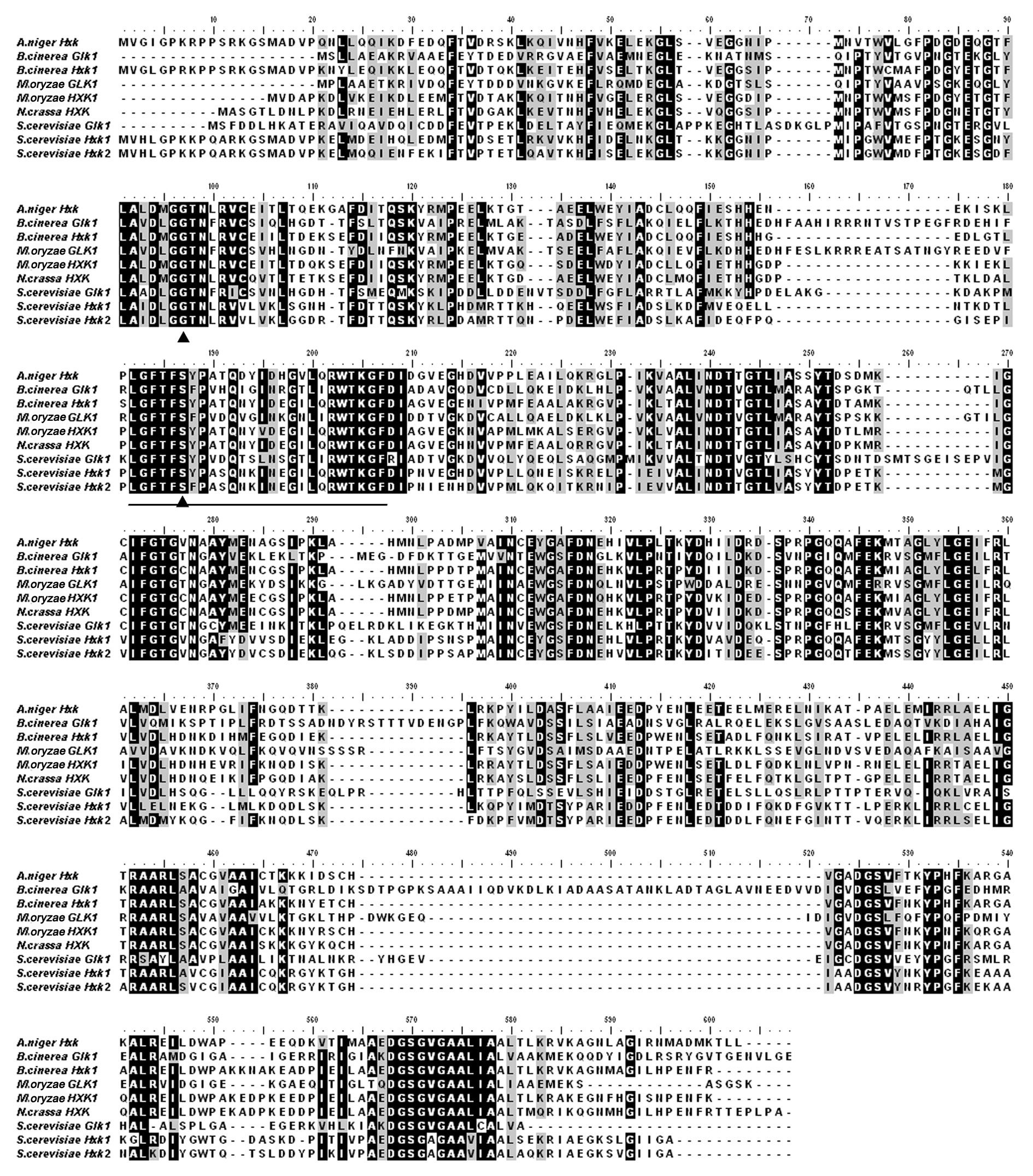

Supplement: Figure S2 — Alignment of MoGLK1 , MoHXK1 and hexokinases and glucokinases in other fungi. The predicted amino acid sequences were aligned using Clustal W. The numbers indicated the amino acid residues. Gaps are indicated by dashes. Identical amino acids are highlighted on a black background, and similar amino acids on a light grey background. The hexokinase signature is marked by a single line. The black triangles indicate the key residues for catalytic activity. (TIF) [file pone.0022809.s002.tif]

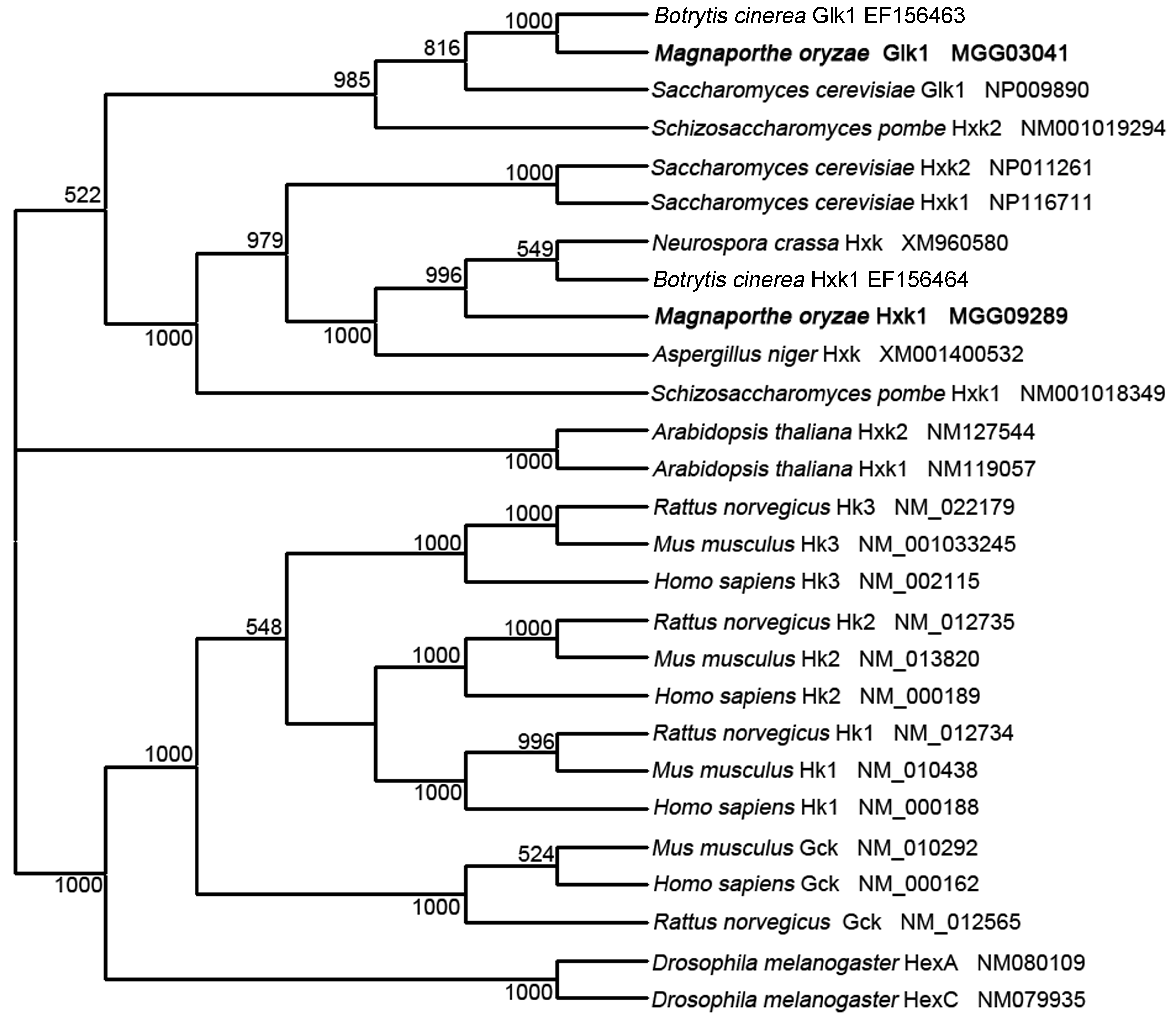

Supplement: Figure S3 — Phylogenetic analysis of MoGLK1 and MoHXK1 . Dendrogram showing the relationship of mammalian, plant, insect and fungal hexose kinases based on amino acid sequences. Sequences were obtained from GenBank. Numbers after genes correspond to Genbank accession numbers. The phylogenetic tree was generated by the neighbor-joining (NJ) method using Mega3.0 Beta. Branch lengths are drawn to scale. (TIF) [file pone.0022809.s003.tif]

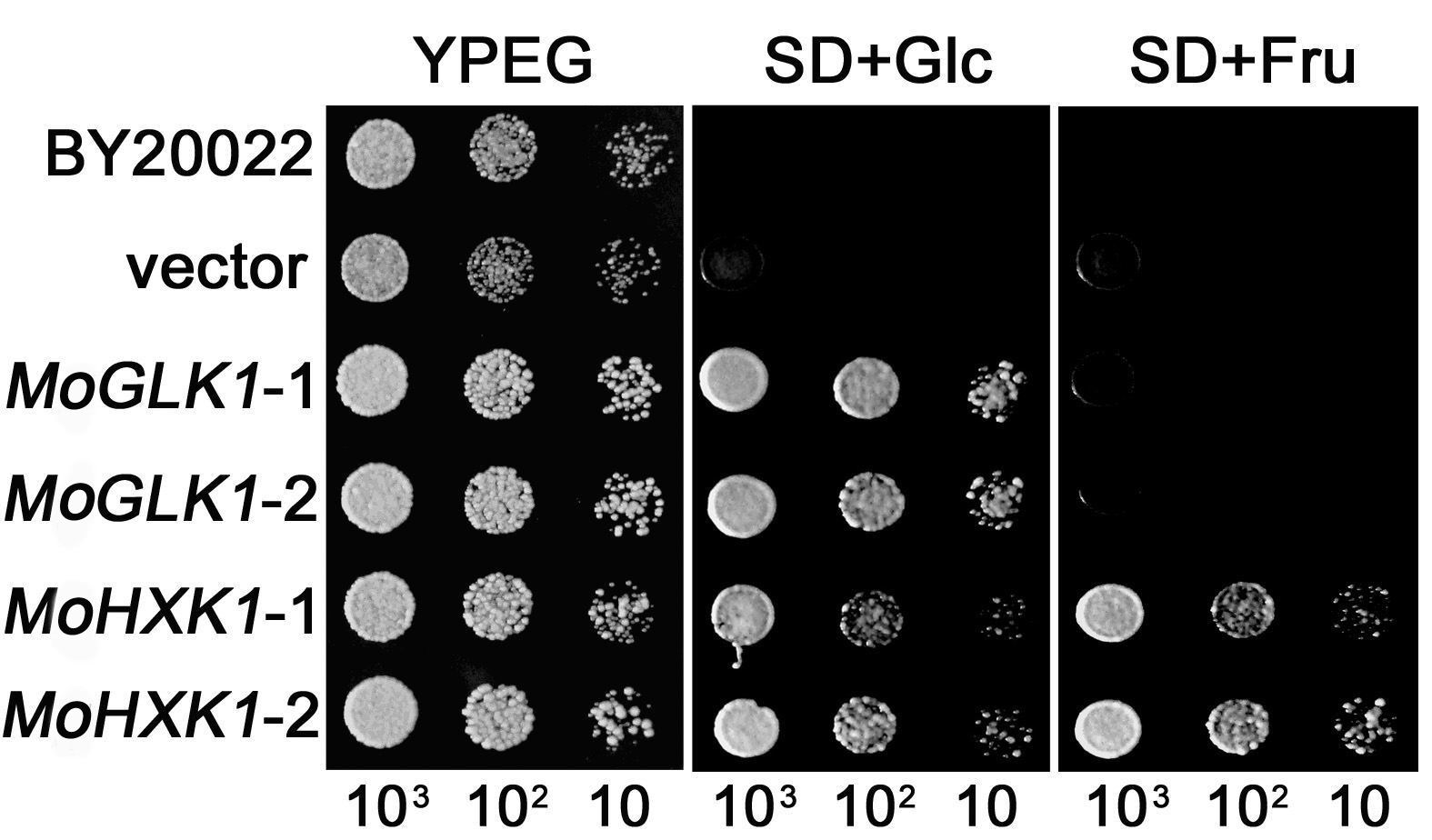

Supplement: Figure S4 — Complementation of S. cerevisiae Δ hxk1 Δ hxk2 Δ glk1 triple mutant. Yeast cells transformed with plasmid pGBKT7 (control) containing MoGLK1 or MoHXK1 were cultured in YPEG medium to an OD600 of ∼1.0 at 30°C. Equal numbers of cells were spotted on YPEG or SD medium plates in the presence of glucose (Glc) or fructose (Fru) as the sole carbon source. Photographs were taken after culturing at 30°C for 4 days. 1 and 2 represent two independent yeast transformants containing MoGLK1 or MoHXK1 constructs. (TIF) [file pone.0022809.s004.tif]

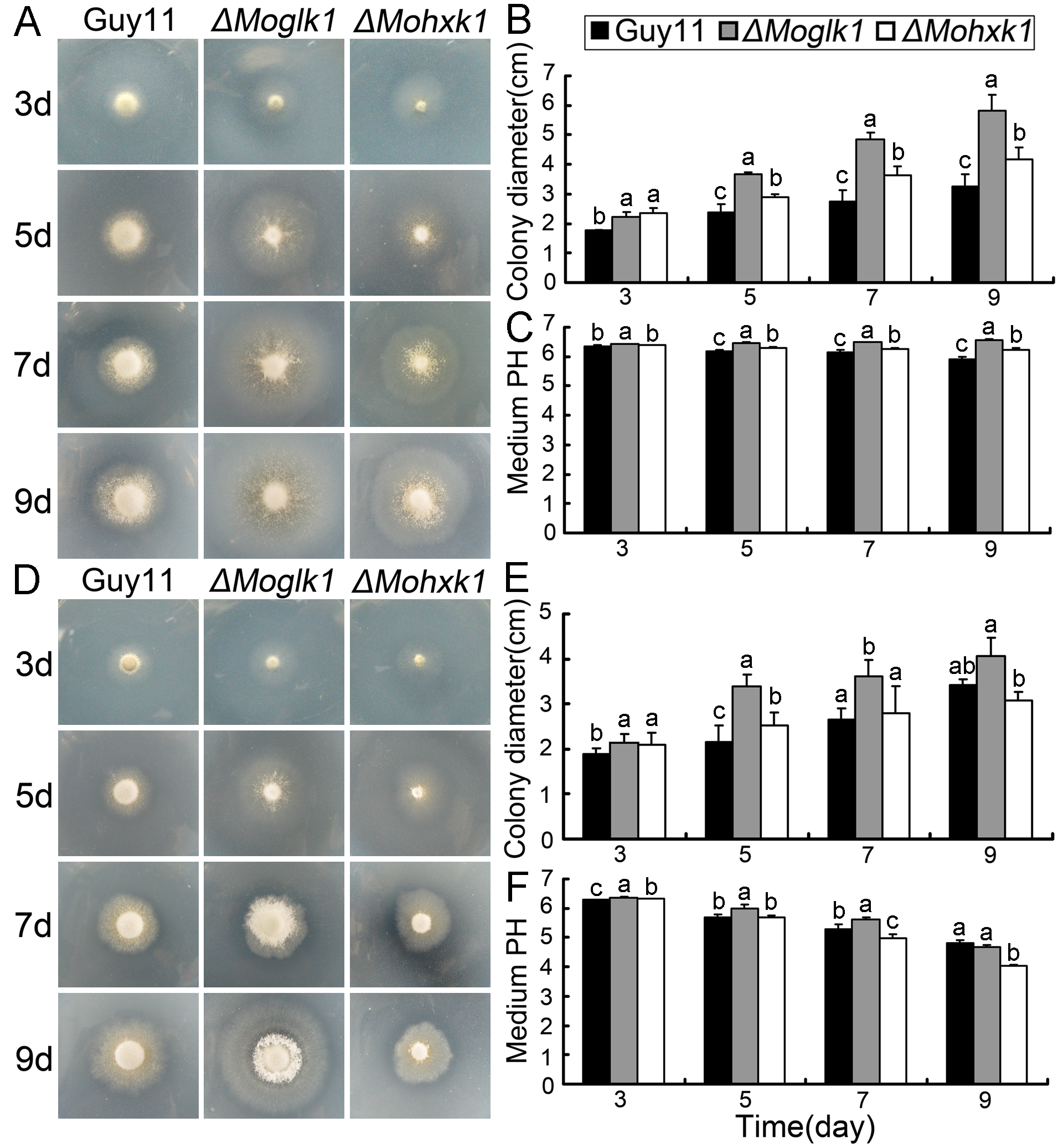

Supplement: Figure S5 — Radial growth of Δ Moglk1 and Δ Mohxk1 mutants on GMM with 1 mM (A, B, and C) and 10 mM (D, E, and F) ammonium. Photographs (A and D) were taken from one transformant of each mutant at indicated days after incubation at 30°C. Colony diameter (B and E) and medium pH (C and F) were measured at the same days. The experiment was performed with three strains for the ΔMoglk1 and ΔMohxk1 mutants, respectively; and three independent replicates provided the same results. Different letter represent difference among (P<0.05) among Guy11 and ΔMoglk1, ΔMohxk1. (TIF) [file pone.0022809.s005.tif]

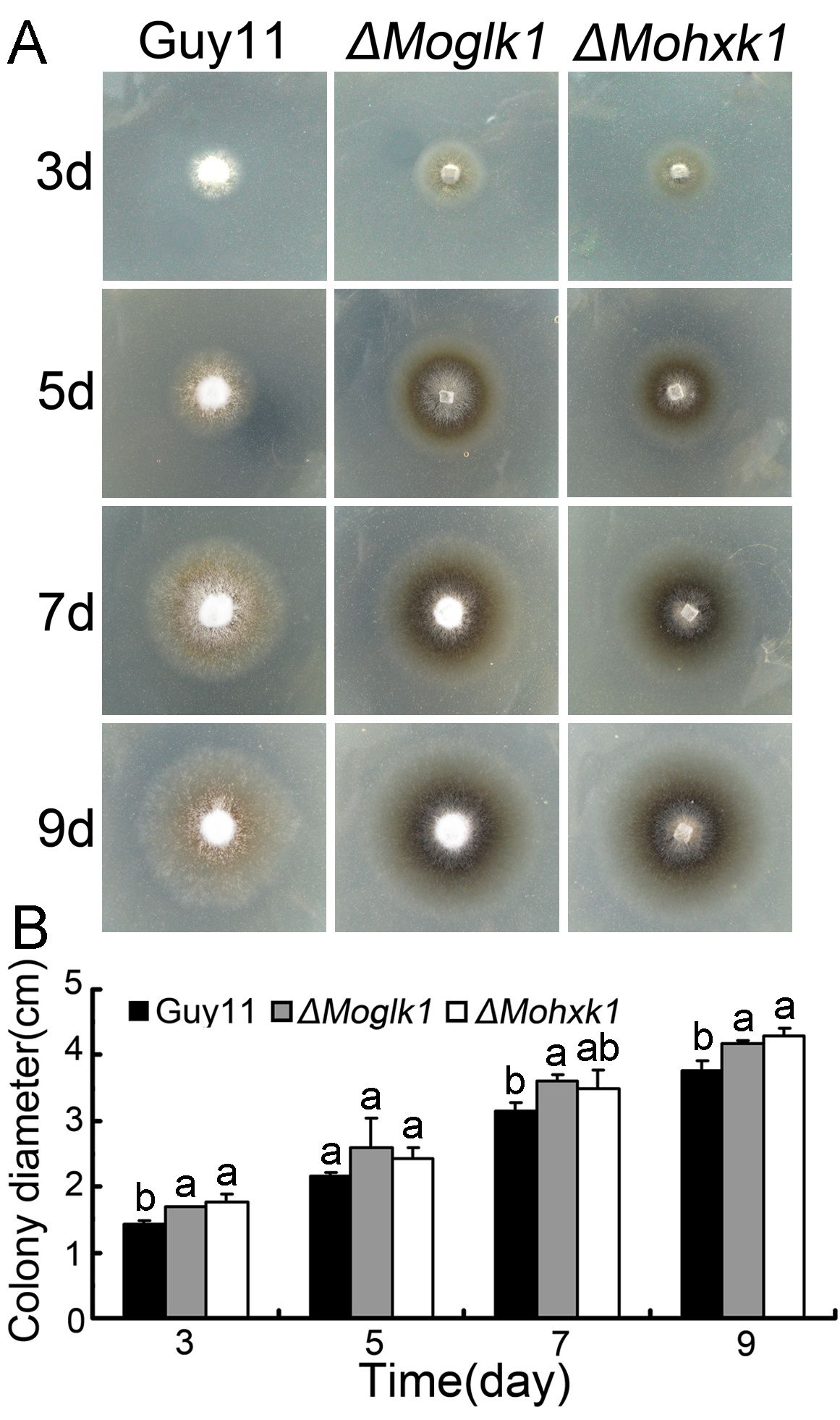

Supplement: Figure S6 — Radial growth of Δ Moglk1 and Δ Mohxk1 mutants on GMM with 100 mM glutamate. Photographs (A) were taken from one transformant of each mutant at indicated days after incubation at 30°C. Colony diameter (B) were measured at the same days. The experiment was performed with three strains for the ΔMoglk1 and ΔMohxk1 mutants, respectively; and three independent replicates provided the same results. Different letter represent difference among (P<0.05) among Guy11 and ΔMoglk1, ΔMohxk1. (TIF) [file pone.0022809.s006.tif]
