# Supplementary material for: The Function of MoGlk1 in Integration of Glucose and Ammonium Utilization in Magnaporthe oryzae
Source: PLoS One. 2011 Jul 27;6(7):e22809. doi: 10.1371/journal.pone.0022809 (PMC3144931; doi:10.1371/journal.pone.0022809)
Supplement: Table S1 — Primer numbers and sequences used in this work. (DOC) [file pone.0022809.s007.doc]

**Table S1**. Primer numbers and sequences used in this work.

| Primer No. | Oligonucleotide sequence |
| --- | --- |
| FL474  FL475  FL640  FL641  FL642  FL863  FL864  FL985  FL990  FL993  FL998  FL1077  FL1078  FL1079  FL1080  FL1359  FL1360  FL1452  FL1979  FL1980  FL2026  FL2027  FL2028  FL2029  FL2500  FL2501  FL4362  FL4363  FL7272  FL7273  FL8986  FL8987  FL8988  FL8989  FL8990  FL8991  FL8992  FL8993  FL8994  FL8995  FL8996  FL8997  FL9509  FL9510  FL9511  FL9512 | 5’-TCGACGTCCGAAAGGATCTGT-3’  5’-ACTCCTGCTTCGAGATCCACATC-3’  5’-TTT**GAATTC**ATGCCTTTGGCAGCAGAGACA-3’  5’-TTT**CTGCAG**CTACTTCGACCCCGAAGCAC-3’  5’-TTT**GAATTC**ATGGTGGACGCTCCGAAGGAT-3’  5’-CAGGACAAGCTCAACTTGGTTC-3’  5’-GAAGCTCAGCTCAGAGGTTGG-3’  5’-GCAATGAACAGAACATCATGG-3’  5’-GGTATGGGAATCATCGACAGC-3’  5’-CAACCCAACGACTATCGGGACG-3’  5’-TGGCATCCCATGTATTCCGG-3’  5’-CAAACTGTCACC**CCCGGG**GGTGCCTGTTCAGTATTCTC-3’  5’-GAACAGGCACC**CCCGGG**GTGACAGTTTGTCCCTCAAC-3’  5’-CAATACTGTCT**CCCGGG**CTTGAGGGGTCTCGAGGTC-3’  5’-GACCCCTCAAG**CCCGGG**AGACAGTATTGGAAGTCATGAG-3’  5’- GCAACTTGATCCGGTGGACC-3’  5’- CTGAAGGGCCCAAACTAAGG-3’  5’-TTT**GTCGAC**TCACTTAAAGTTCTCTGGGTTGC-3’  5’-CTGTCTGCATCTCGAGATCTAC -3’  5’-GGAGGCGAGGATGTCGACAC -3’  5’-CTGAAGTTTGTGTCGCCAAGATC-3’  5’-GATCTTGGCGACACAAACTTCAG-3’  5’-CACGGGGAAGGCGAAGGTGAAAC-3’  5’-GTTTCACCTTCGCCTTCCCCGTG-3’  5’-TTT**AAGCTT**GGAGGTCAACACATCAATG -3’  5’-TTT**GAATTC**ATCGATGCTTCGGTAGAATAGG-3’  5’-CCATGTACCCTGGTCTTTCG-3’  5’-TTCGAGATCCACATCTGCTG-3’  5’-AGGGCGTTGACATTAACTGG-3’  5’-CGAAGGGAGTGAACTTCTCG-3’  5’-CGAGGAGAATGGTGGAAGC-3’  5’-GACTAATGATGGTGCCCTCG-3’  5’-ATCCTCTTCCTCATGAACCTG-3’  5’-CTTGCTGACCTCCTGGGTCT-3’  5’-GAGCTACTCTACTCGTTCTAC-3’  5’-TGGCATTCCAAACCCAATG-3’  5’-GGTCAGGACGACTTGGTATC-3’  5’-GACGTTGAAGTCCCAGAGAC-3’  5’-TTGAGTACCTGCAGCTTCTC-3’  5’-TCTTGTGCCTGTGACAGTAC-3’  5’-AGTGGCGAGATCACGATCCA-3’  5’-CCTTGCCCTGATTGTCCAAG-3’  5’-AATTCAGGGGCTACGCCTAT-3’  5’-CCCAAAAGCACCAGCAGAAG-3’  5’-ACATGGAAGTGGAGGTGGAG-3’  5’-CAAAAAGTACCTGCCCCTGA-3’ |

Restriction sites are indicated in bold.
